# Regulation of polyamine interconversion enzymes affects α-Synuclein levels and toxicity in a Drosophila model of Parkinson’s Disease

**DOI:** 10.21203/rs.3.rs-6648986/v1

**Published:** 2025-05-15

**Authors:** Bedri Ranxhi, Zoya R. Bangash, Zachary M. Chbihi, Zaina Qadri, Nazin N. Islam, Sokol V. Todi, Peter A. LeWitt, Wei-Ling Tsou

**Affiliations:** Wayne State University; Wayne State University; Wayne State University; Wayne State University; Wayne State University; Wayne State University; Henry Ford Health Systems; Wayne State University

**Keywords:** α-Synuclein, Polyamines, Spermine oxidase, Spermidine/spermine N1-acetyltransferase 1, Neurodegenerative Diseases

## Abstract

Parkinson’s Disease (PD) is a prevalent neurodegenerative disorder characterized by the accumulation and aggregation of α-synuclein as a defining pathological hallmark. Misfolding and aggregation of α-synuclein disrupt cellular homeostasis, hinder mitochondrial function, and activate neuroinflammatory responses, ultimately resulting in neuronal death. Recent biomarker studies have reported a significant increase in the serum concentrations of three L-ornithine-derived polyamines, correlating with PD progression and its clinical subtypes. However, the precise role of polyamine pathways in PD pathology remains poorly understood. In this study, we explored the impact of modifying polyamine-interconversion enzymes (PAIE) on the α-synucleinopathy phenotype in a *Drosophila melanogaster* model of Parkinson’s Disease (PD). We assessed key degenerative features, including lifespan, locomotor function, tissue integrity, and α-synuclein accumulation. We found that PAIEs play a critical role in modulating α-synuclein toxicity in the PD model. Knockdown of ornithine decarboxylase 1 (ODC1), spermidine synthase (SRM), and spermine oxidase (SMOX) mitigates α-synuclein toxicity, whereas suppression of spermidine/spermine N1-acetyltransferase 1 (SAT1) and spermine synthase (SMS) exacerbates it. Furthermore, the overexpression of SAT1 or SMOX significantly lowers α-synuclein toxicity, emphasizing their potential involvement in PD. These results highlight the importance of polyamine pathways in PD, where PAIEs are essential in managing α-synuclein toxicity, providing a new perspective on targeting PD’s fundamental pathology.

## Introduction

Parkinson’s Disease (PD) is a progressive neurodegenerative disorder affecting millions of mid-life individuals and is characterized by a decline in motor function (including slowed movements, tremors, and cognitive decline)^1,2^. The primary risk factor is increasing age, although environmental factors and genetics may also play a role^3–5^. The motor disorder of PD involves the degeneration of a specific population of neurons located in the substantia nigra which project to the striatum and generate dopamine^6–8^. While most cases of PD appear sporadic^9^, some cases arise from various gene mutations^5^, the most common being *LRRK2*^10^, *and GBA1*^11^. Additionally, more than 2 dozen gene mutations have been associated with causation or enhanced risk for PD^10,12^. While PD might have multiple etiologies, a central hallmark of the disease is the pathological accumulation of α-synuclein (α-Syn)^13–15^, a small, soluble protein encoded by the *SNCA* gene. α-Syn plays an essential role in synaptic function^16^ and neurotransmitter release^17^.

In PD, α-Syn undergoes structural changes, misfolding, and aggregation into insoluble fibrils^18^. These α-Syn aggregates, commonly seen in PD brain tissue in circular structures known as Lewy bodies^19^, accumulate over time in the progressive disease and also in aging individuals as an incidental finding^20^. Abnormal α-Syn aggregates interfere with critical cellular processes, including mitochondrial dynamics^21^, proteostasis^22^, and endo-lysosomal membrane integrity^23^. Ultimately, these processes result in selective neuronal damage and death. Thus, there is a need for mechanistic studies to further investigate disease-related α-Syn aggregation and its role in PD progression.

The aggregation process of α-Syn, driven by elevated α-Syn protein levels, is influenced by both genetic^4,24–29^ and environmental factors^30–32^. Recent biomarker studies suggest that the concentration of polyamines (PAs) is altered in PD^33,34^. PAs are essential organic polycations that are evolutionarily conserved^35^ across diverse organisms, from yeast and bacteria to plants and mammals. They are ubiquitous in cells and play critical roles in numerous cellular processes, including cell growth^36^, nucleic acid synthesis^37,38^, ion transport^37,38^, and apoptosis^39,40^. Dysregulation of PA homeostasis can lead to various adverse outcomes^41^ in humans, culminating in disease and pathology; multiple reports link altered PA metabolism to various types of cancer^42–44^, cardiovascular disease^45^, and neurodegeneration^46–48^. Serum biomarker studies in PD identified an increase in three L-ornithine (ORN)- derived PAs, putrescine (PUT), spermidine (SPD), and spermine (SPM), in early-stage PD patients, all of which correlated with the progression of PD and its clinical subtypes^34^. Whether this correlation was related to the elevated α-Syn protein levels is unknown.

PAs can have a dual role in neurodegenerative diseases, functioning as facilitators that preserve neuronal integrity^49^ by promoting autophagy to clear toxic proteins like α-Syn^50^ and reduce oxidative stress, thus supporting neuronal survival. Conversely, PAs contribute to neuronal damage through their catabolism, which produces reactive oxygen species such as H_2_O_2_ and acrolein^51,52^, leading to oxidative stress, inflammation, and excitotoxicity^53^ that harm neurons^54^. The balance of PA pathways underscores their critical role in PD pathology^55,56^. However, it remains unclear whether elevated PA concentrations directly exacerbate PD pathology by promoting oxidative stress and inflammation, or if they are a secondary effect of disease progression, reflecting compensatory mechanisms to mitigate neuronal damage.

The intracellular homeostasis of ORN, PUT, SPD, and SPM is meticulously maintained through synthesis, degradation, and export^57,58^. PA biosynthesis converts ORN into PUT and with further incorporation of aminopropyl groups into SPD and SPM through specific polyamine interconversion enzymes (PAIEs)^35^. These include PA anabolic and catabolic enzymes^59^. PA anabolic enzymes, such as ornithine decarboxylase (ODC1), spermidine synthase (SRM), and spermine synthase (SMS), facilitate the biosynthesis of PAs^57,58^. This process involves a series of decarboxylation reactions followed by aminopropylation^60^. PA catabolism is a more complex process in which PAs are broken down into their precursors and is facilitated by a distinct group of PAIEs that include spermine oxidase (SMOX), spermidine/spermine N^1^-acetyltransferase (SAT1), and N^1^-acetylpolyamine oxidase (PAOX)^61^. Catabolic PAIEs are involved in acetylation and oxidation processes^62,63^. Additionally, selective transporters mediate the translocation of PAs and their byproducts across cellular membranes. The Na^+^-independent transporter SLC7A2^64^ supports PA synthesis by facilitating the uptake of cationic amino acids and ornithine (ORN). Furthermore, ATP-dependent transporters ATP13A2 and ATP13A3^65^ are essential for PA trafficking within the endo-/lysosomal system, ensuring efficient distribution and homeostasis of PAs in cellular compartments. These mechanisms highlight the complex regulation of PA dynamics in cells. Investigating PA pathways – including metabolites, interconversion enzymes, and transporters^66,67^ – may provide a better understanding of PD's pathogenic mechanisms, as indicated by the serum biomarker^33,34^ and related findings^55^.

Here, we utilized *Drosophila melanogaster*to investigate the significance of PA pathway perturbation in PD pathology, modeled through the neuronal overexpression of human wild-type α-Syn^68,69^. Our aim was to determine whether targeted PA metabolism could affect α-Syn stability and impact disease progression. We observed that the regulation of PAIEs significantly affects α-Syn toxicity. We identified the PA catabolic enzymes SAT1 and SMOX as critical factors in PD, as they influenced α-Syn protein levels and its effects in *Drosophila.* Our findings provide novel mechanistic insights into a PD model, using α-Syn pathology as a readout to advance biomarker research and set the stage for PA-targeted therapies.

## Materials and Methods

### Drosophila Stocks and Maintenance

Stock numbers and genotypes of all flies are listed in Table 1. Publicly available stocks were obtained from the Bloomington Drosophila Stock Center (BDSC) or the Vienna Drosophila Resource Center (VDRC). Flies overexpressing UAS-DmSAT1 and UAS-DmSMOX were generated in our laboratory. cDNA of DmSAT1 (CG4210) and DmSMOX (CG7737) with an in-line HA tag was synthesized by GenScript (Piscataway, NJ), cloned into the pWaliumlO.moe plasmid, and injected into fly embryos for insertion into the attP2 site. Genomic DNA was extracted and sequenced to confirm line integrity and identity. Flies were reared in 5 mL of standard cornmeal fly medium supplemented with 2% agar, 10% sucrose, 10% yeast, and appropriate preservatives, under a 25°C incubator at 40% humidity with a 12/12-hour light/dark cycle. In all experiments, food vials were replaced every two to three days.

#### Longevity assay

Approximately 20 adult flies, matched by age and separated by sex within 48 hours of eclosion as adults from their pupal cases, were collected per vial and maintained on standard cornmeal fly medium at 25°C. Flies were transferred to fresh food vials every 2–3 days, and mortality was monitored daily until all flies had died. Total fly numbers are indicated in each figure. Survival data were analyzed using the log-rank test in GraphPad Prism (San Diego, CA, USA).

#### Motility assay

Negative geotaxis was assessed through a modified Rapid Iterative Negative Geotaxis (RING) assay^68,70,71^ involving groups of at least 100 flies. Vials with 20 flies each were tapped to force them to settle at the bottom, and their climbing responses were captured with photographs taken 3 seconds afterward. Weekly records of the average performance from five consecutive trials were maintained, with flies kept on standard food between tests. Positions of the flies in specified vial zones were quantified as percentages using RStudio (Boston, MA, USA), following the methodology described in our previous study^68^.

#### CD8-GFP fluorescence measurements

All flies analyzed in this study were heterozygous for both the driver (GMR-Gal4) and transgenes (UAS-CD8-GFP and UAS-RNAi). Progeny were collected at eclosion and aged for 14 and 28 days. At these intervals, fly heads were dissected and imaged for GFP fluorescence using an Olympus BX53 microscope with a 4X objective and a DP72 digital camera. The fluorescence intensity was quantified using ImageJ, as previously described^72,73^. Statistical analysis of GFP expression was performed using ANOVA in GraphPad Prism 9 (San Diego, CA, USA). All groups had n ≥ 28 flies.

#### Western blots

Fourteen fly heads (7 males, 7 females) per replicate were homogenized in hot lysis buffer (50 mM Tris pH 6.8, 2% SDS, 10% glycerol, 100 mM dithiothreitol), sonicated, boiled for 10 minutes, and centrifuged at maximum speed for 10 minutes. Protein lysates from at least three replicates were analyzed by Western blotting using 4–20% Mini-PROTEAN^®^ TGX^™^ Gels (Bio-Rad) and transferred to 0.2 μm PVDF membranes. After blocking in 5% milk/TBST, membranes were incubated overnight at 4°C with primary antibodies: mouse anti-α-Syn (4B12) (1:1000, Sigma-Aldrich) and anti-HA (1:1000, Cell Signaling Technology), followed by secondary peroxidase-conjugated antibodies (1:5000, Jackson ImmunoResearch) for 1 hour at room temperature. Signal detection used EcoBright Pico/Femto HRP substrates (Innovative Solutions), imaged on a ChemiDoc system (Bio-Rad). PVDF membranes were stained with 0.1% Direct Blue 71 for total protein visualization, and band intensities were quantified using ImageLab software (Bio-Rad).

### Statistics

For Western blots, the levels of α-Syn were normalized to Direct Blue staining and compared against control groups. Prism 9 (GraphPad) was used for data visualization and statistical analyses, with all statistical methods detailed in the figure legends.

## Results

### Expression of human α-Syn leads to shortened lifespan and motor dysfunction in Drosophila

**Figure 1:**
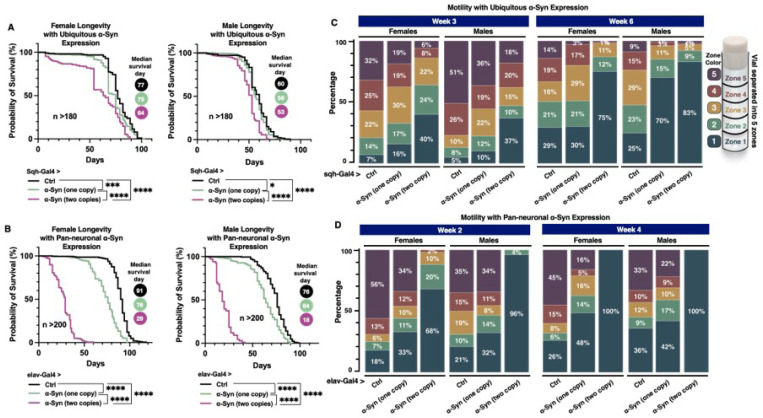
Longevity and motility analyses of flies ubiquitously or pan-neuronally expressing α-Syn **(A, B)** Longevity curves of adult female (left) and male (right) flies expressing zero (black), one (green), or two (pink) copies of α-Syn throughout development and adulthood, driven by **(A)** sqh-Gal4 and **(B)**elav-Gal4. Median survival days are indicated to the right of each panel. Statistical significance was assessed using log-rank tests: ns (not significant), * (p < 0.05), ** (p < 0.01), *** (p < 0.001), **** (p < 0.0001). **(C, D)** Motility analysis (RING assay) of flies with **(C)**ubiquitous and **(D)** pan-neuronal α-Syn expression, with the week of measurement indicated at the top. Each vial was divided into five zones, with different colors representing each zone. Flies were photographed, and the number of flies in each zone was counted and normalized to the total number of flies. The percentage of flies in each zone is shown in the figure.

We employed overexpression of human wild-type α-Syn as a *Drosophila* model to investigate the impact of PA pathway modulation in PD. As an initial step, we validated the model by assessing whether α-Syn expression induces neurodegenerative phenotypes when driven ubiquitously or specifically in neurons. As shown in Fig. 1A, ubiquitous expression of α-Syn using the sqh-Gal4 driver resulted in a dose-dependent reduction in lifespan in both male and female flies, with two copies of the transgene reducing median lifespan to 64 days in females and 53 days in males. A more pronounced effect was observed with pan-neuronal expression of α-Syn driven by elav-Gal4, as illustrated in Fig. 1B. Flies carrying two copies of α-Syn had a median lifespan of 29 days in females and 18 days in males, compared to 76 days in females and 64 days in males with only one copy. These results confirm that α-Syn dosage strongly influences survival, particularly when expressed in neurons. Next, we examined a secondary aspect of fly physiology by assessing fly mobility through the Rapid Iterative Negative Geotaxis (RING) assay^70^. This assay was conducted three and six weeks post-eclosion of flies ubiquitously expressing α-Syn (Fig. 1C). At week three, compared to control flies that contained the Gal4 driver in the absence of α-Syn, a smaller proportion of α-Syn-expressing flies reached zones 4 and 5, the highest tiers of the motility index. This decline in motility was also dose-dependent, with flies carrying two copies of the α-Syn transgene exhibiting a more pronounced impairment in both sexes. Notably, sex-specific differences emerged at week six, with a higher proportion of male flies expressing one or two copies of α-Syn remaining in zone 1 compared to their female counterparts, indicating more severe locomotor deficits. These sex-specific differences mirror observations in human populations, where PD is more common in men, with around 65% of patients being male^74,75^.

We also conducted the same RING assay on flies overexpressing α-Syn pan-neuronally (Fig. 1D). Due to the early mortality observed in flies with pan-neuronal α-Syn expression, we performed assays at two and four weeks post-eclosion. Pan-neuronal expression of α-Syn led to markedly greater locomotor impairments compared to flies with ubiquitous α-Syn expression. Notably, at both two and four weeks, 96–100% of male flies carrying two copies of the α-Syn transgene remained confined to zone 1 at the bottom of the vial, highlighting the severity of the phenotype. Sex-specific differences were observed as early as week two in flies pan-neuronally expressing two copies of the α-Syn transgene, with the proportion of males remaining in zone 1 being approximately 30% higher compared to females. We conclude that expression of α-Syn in flies leads to reduced motility and longevity in a dose-dependent manner. These baseline data establish the α-Syn overexpression fly model as a valuable tool for investigating the relationship between PA metabolism and α-Syn toxicity.

### Targeting of PA interconversion enzymes (PAIE) modulates α-Syn toxicity in Drosophila

**Figure 2:**
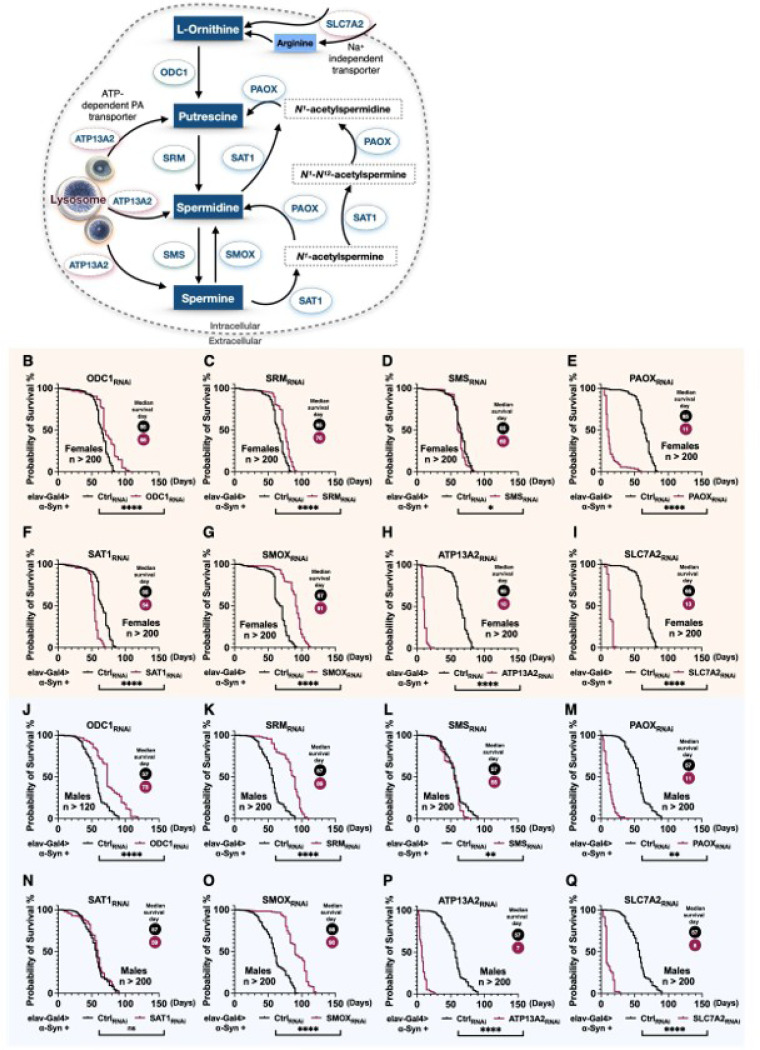
Knockdown of enzymes in the polyamine pathway alters longevity in the α-Syn *Drosophila* model **(A)**Schematic of the polyamine pathway and the associated enzymes. **(B-Q)**Longevity analysis of neuronal knockdown of individual polyamine pathway enzymes in the α-Syn *Drosophila* model. Panels **(B-I)** show lifespan data for female flies, while panels **(J–Q)** present data for male flies. Statistical significance was determined using log-rank tests: ns (not significant), * (p < 0.05), ** (p < 0.01), *** (p < 0.001), **** (p < 0.0001).

Given the elevated levels of PAs observed in the serum of PD patients^33,34^, we sought to determine whether regulating the PA pathway could influence disease-related phenotypes in our PD model. To address this, we examined the effects of PA pathway modulation in our *Drosophila* model with pan-neuronal expression of α-Syn. As illustrated in Fig. 2A, the PA pathway constitutes a tightly regulated metabolic network comprising anabolic and catabolic interconversion enzymes, along with transporters that collectively maintain PA homeostasis. To assess how individual PAIE influence α-Syn–driven neurodegeneration, we performed RNAi-mediated neuronal knockdowns of *Drosophila* orthologs of PAIEs and PA transporters. In our longevity assays, knockdown of ODC1 (Fig. 2B, 2J), SRM (Fig. 2C, 2K), or SMOX (Fig. 2G, 2O) significantly extended lifespan in both female and male α-Syn flies compared to RNAi controls. Notably, knockdown of SAT1 reduced lifespan in female flies (Fig. 2F) but not in males (Fig. 2N), suggesting a sex-specific effect. Additionally, neuronal knockdown of PAOX, ATP13A3, or SLC7A2, regardless of α-Syn expression, led to pronounced developmental abnormalities such as unexpanded wings and impaired leg mobility, resulting in early lethality (Fig. 2E, 2H, 2I, 2M, 2P, 2Q). These findings indicate that these genes are essential for normal development and function independently of α-Syn–associated toxicity. Due to these developmental defects, proper lifespan comparisons under α-Syn expression could not be assessed for these knockdowns. In summary, the lifespan extension observed following ODC1, SRM, or SMOX knockdown highlights the potential protective role of modulating specific PAIE pathways in the context of α-Syn–induced pathology.

**Figure 3:**
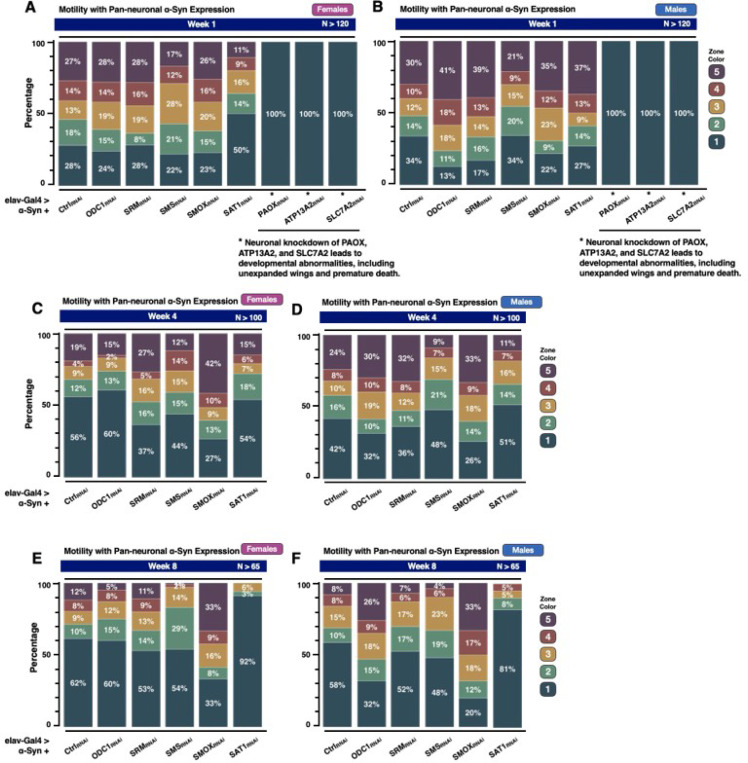
Knockdown of enzymes in the polyamine pathway impact motility in the α-Syn *Drosophila* model **(A-F)** RING assay results showing the effects of neuronal knockdown of individual polyamine pathway enzymes in the α-Syn *Drosophila* model at week 1 (**A, B**), week 4 (**C, D**), and week 8 (**E, F**), with female flies on the left and male flies on the right. In **(A, B)**, asterisks indicate that flies with neuronal knockdown of PAOX, ATP13A2, and SLC7A2 were assessed only at week 1, as these flies exhibited severe developmental abnormalities, including unexpanded wings, and died within three weeks.

Next, we examined whether modulation of PAIEs affects the motility of the α-Syn Drosophila model using the RING assay (Fig. 3). In week one, knockdown of SMS and SAT1 impaired climbing ability in female flies, with fewer individuals reaching the higher zones (4 and 5); notably, SAT1 knockdown resulted in 50% of flies remaining in zone 1 (Fig. 3A). In male flies, knockdown of ODC1, SRM, SMOX, or SAT1 initially enhanced climbing performance (Fig. 3B), with a greater proportion reaching zone 5 and fewer remaining in zone 1. As observed in the longevity experiments (Fig. 2), knockdown of PAOX, ATP13A2, or SLC7A2 caused severe developmental abnormalities. These flies displayed a complete inability to climb and remained in zone 1 at the bottom of the vial (Fig. 3A, 3B).

By the fourth week, female flies with knockdown of SRM or SMOX showed a marked improvement in mobility, as indicated by a higher proportion of flies reaching zone 5 and fewer remaining in zone 1 (Fig. 3C). In contrast, knockdown of ODC1, SMS, or SAT1 resulted in reduced mobility, with fewer females reaching zone 5. In males, knockdown of ODC1, SRM, or SMOX (Fig. 3D) enhanced locomotor performance, with a greater percentage of flies reaching zone 5 compared to controls. Conversely, knockdown of SMS or SAT1 led to decreased motility, with fewer flies reaching zone 5 and more remaining in zone 1. By the eighth week, SMOX knockdown continued to promote improved motor function, with 33% of both female (Fig. 3E) and male (Fig. 3F) flies reaching zone 5, compared to only 12% and 8% in the respective control groups. Interestingly, ODC1 knockdown led to improved mobility in males only (Fig. 3F), with 26% reaching zone 5 and 32% remaining in zone 1. Knockdown of SAT1 consistently impaired locomotor function in both sexes, with the majority of flies confined to zone 1 (92% in females and 81% in males). Knockdown of SMS also reduced motility, though to a lesser extent, with only 1% of females and 4% of males reaching zone 5. Together, these findings indicate that SMOX knockdown significantly improves locomotor outcomes in the α-Syn model, while SAT1 knockdown consistently worsens them.

#### PAIE regulates fly eye integrity in the context of α-Syn-Induced Toxicity

**Figure 4:**
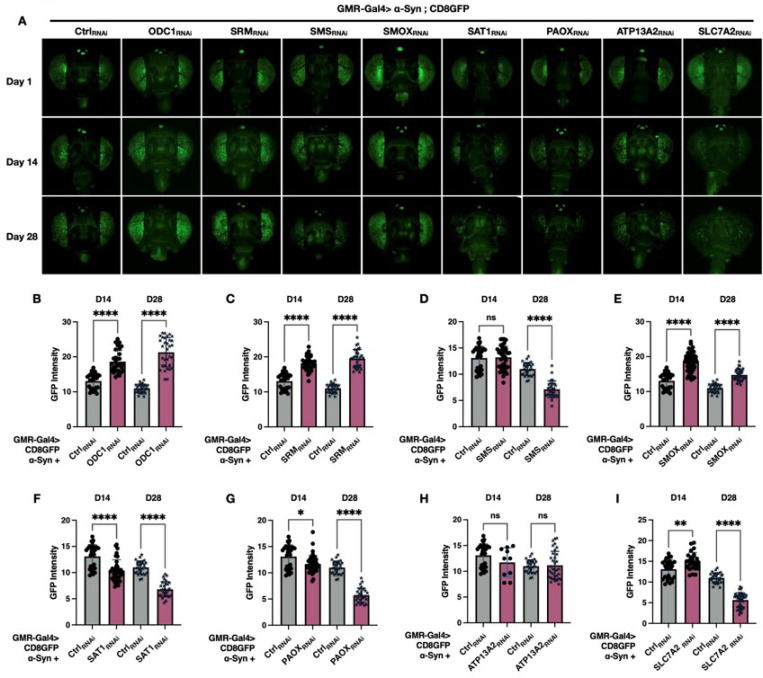
Polyamine pathway enzyme knockdown modulates eye integrity in the α-Syn *Drosophila* model, assessed by CD8-GFP fluorescence. **(A)** Representative GFP images of fly heads co-expressing CD8-GFP, α-Syn, and RNAi targeting polyamine pathway enzymes in the eye. Female fly head images were gathered on days 1, 14, and 28 post-eclosion. **(B–I)**Quantiflcation of GFP fluorescence intensity at days 14 and 28 using ImageJ. Sample size: N ≥ 15 per condition. Statistical analysis was performed using Brown-Forsythe and Welch ANOVA tests. Significance levels: ns (not significant), * (p < 0.05), ** (p < 0.01), *** (p < 0.001), **** (p < 0.0001).

We observed that neuronal PAIE knockdown influences longevity and motility phenotypes in the α-Syn model. To investigate whether these effects are associated with cellular-level changes in neuronal integrity, we utilized the *Drosophila* eye, a well-established system for studying neurodegeneration and cellular toxicity. Each ommatidium of the compound eye contains a cluster of photoreceptor neurons. By expressing a membrane-tagged fluorescent marker, CD8-GFP, in these photoreceptors, we were able to visualize cellular architecture and assess neuronal integrity *in vivo*^72,76,77^. This model provides a robust and quantifiable platform for evaluating α-Syn–induced toxicity in response to PAIE modulation. In this context, toxicity is reflected by the degeneration of internal ommatidial components, leading to photoreceptor cell loss and reduced GFP fluorescence^72^. Conversely, enhanced fluorescence indicates preserved eye structure and improved neuronal integrity^78^. Figure 4A represents the GFP photos of the fly eyes, significantly enhanced GFP signal was shown in the eyes of ODC1 _RNAi_, SRM _RNAi_, and SMOX _RNAi_ at days 1, 14, and 28. Quantification of GFP intensity (Fig. 4B-I) confirmed that knockdowns of ODC1 (Fig. 4B), SRM (Fig. 4C), or SMOX (Fig. 4E) at days 14 and 28 led to a notable increase in fluorescence compared to background controls. In contrast, flies co-expressing α-Syn with either SAT1_RNAi_ (Fig. 4F) or PAOX_RNAi_ (Fig. 4G) at days 14, 28, as well as those with SMS_RNAi_ (Fig. 4D) at day 28 exhibited significantly reduced GFP intensity compared to the controls. Moreover, while knockdown of PA transport enzyme ATP13A2 did not alter GFP fluorescence (Fig. 4H), knockdown of the sodium-independent transporter SLC7A2 led to a significant GFP reduction at day 28 (Fig. 4I). Overall, these results indicate that knockdown of ODC1, SRM, and SMOX enhances cellular integrity in the α-Syn model, whereas knockdown of SAT1, SMS, PAOX, or SLC7A2 exacerbates cellular toxicity. Collectively, these findings underscore the significance of PAIE modulation in mitigating α-Syn-induced toxicity.

#### PAIE knockdowns affect α-Syn protein levels

**Figure 5:**
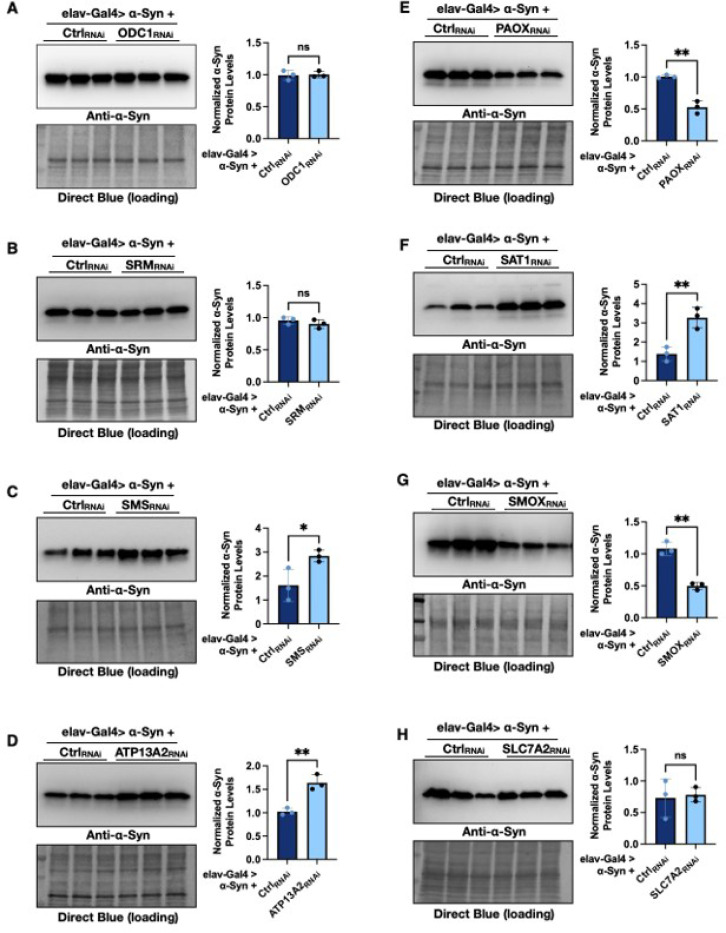
Polyamine pathway enzyme knockdown modulates α-Syn protein levels **(A-H)** Western blot analysis of α-Syn protein levels in flies with pan-neuronal expression of α-Syn following RNAi-mediated knockdown of specific polyamine pathway enzymes. Statistical significance was assessed using an unpaired two-tailed Student’s t-test. Significance levels are indicated as follows: ns (not significant), * (p < 0.05), ** (p < 0.01), *** (p < 0.001), **** (p < 0.0001).

To further understand how PAIE modulation influences α-Syn-induced toxicity, we next examined whether changes in PAIE expression affect α-Syn protein levels. Since α-Syn accumulation and aggregation are central features of PD pathology, we assessed α-Syn protein abundance in flies with pan-neuronal expression of α-Syn and RNAi-mediated knockdown of individual PAIE genes (Fig. 5 with quantification on the right). We observed significant increases in α-Syn protein levels following knockdown of SMS (Fig. 5C), ATP13A2 (Fig. 5D), and SAT1 (Fig. 5F). In contrast, α-Syn protein levels were reduced when PAOX (Fig. 5E) or SMOX (Fig. 5G) was knocked down. Knockdown of ODC1 (Fig. 5A), SRM (Fig. 5B), or SLC7A2 (Fig. 5H) did not result in notable changes in α-Syn levels. Together, these findings suggest that individual PAIE enzymes differentially regulate α-Syn protein homeostasis, with knockdown of PAOX and SMOX reducing α-Syn accumulation, while the knockdowns of SMS, SAT1, or ATP13A2 promote it.

### Overexpression of SAT1 and SMOX mitigate α-Syn-induced toxicity in Drosophila

**Figure 6:**
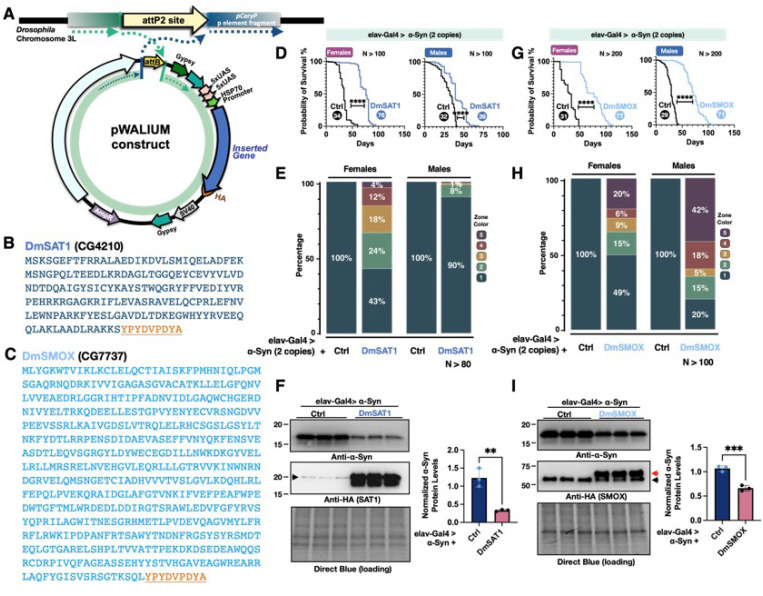
Overexpression of DmSATI and DmSMOX alters disease-related phenotypes in the α-Syn *Drosophila* model. **(A)** A diagram of the cloning strategy used to insert DmSAT1 or DmSMOX into the pWALIUM10.moe vector, with plasmids integrated into the third chromosome of the pCary fly line at the attP2 site; **(B, C)** amino acid sequences of HA-tagged DmSATI (**B**) and DmSMOX (**C**), with the HA tag underlined in orange; **(D, G)** longevity analysis of flies with pan-neuronal expression of two copies of α-Syn, with or without overexpression of DmSATI (**D**) or DmSMOX (**G**), where the numbers in the circles indicate median survival days and statistical significance was determined using log-rank tests (ns: not significant, *p < 0.05, **p < 0.01, ***p < 0.001, ****p < 0.0001); **(E, H)** motility analysis using the RING assay at week 4 in flies expressing two copies of α-Syn, with or without DmSATI (**E**) or DmSMOX (**H**) overexpression; **(F, I)** western blot analysis of α-Syn protein levels in flies with pan-neuronal expression of α-Syn, with or without DmSATI (**F**) or DmSMOX (**I**) overexpression, where the arrowhead in **F** indicates non-specific bands and in I, the red arrow marks SMOX-specific bands and the black arrow denotes non-specific bands; statistical analysis for western blots was performed using an unpaired two-tailed Student’s t-test with significance levels as follows: ns (not significant), * (p < 0.05), ** (p < 0.01), *** (p < 0.001), **** (p < 0.0001).

We have compared longevity, motility, eye integrity, and α-Syn protein levels in the α-Syn *Drosophila* models and discovered that suppressing enzymes in the PA pathway affects α-Syn–mediated toxicity. Given the strong effects we observed with SAT1_RNAi_ and SMOX_RNAi_, we were interested in whether overexpressing these genes would produce the opposite outcome or further support their regulatory roles. To address this, we generated new fly lines carrying UAS-DmSAT1 or UAS-DmSMOX by inserting *Drosophila* SAT1 or SMOX cDNA into the attP2 site on chromosome 3 (Fig. 6A-C). We utilized flies with pan-neuronal expression of two copies of α-Syn to induce a stronger phenotype and tested whether overexpression of DmSATI could rescue it. As expected, DmSATI overexpression significantly extended lifespan compared to control flies (Fig. 6D). Furthermore, DmSATI markedly improved climbing ability, with females showing a more pronounced enhancement than males, as more flies reached the higher zones 4 and 5. Western blot analysis also revealed a reduction in α-Syn protein levels in the presence of DmSAT1 overexpression (Fig. 6F).

Similarly, we tested flies with pan-neuronal expression of both DmSMOX and α-Syn. Intriguingly, despite the protective effects previously observed with SMOX knockdown, overexpression of DmSMOX also significantly extended the lifespan of α-Syn-expressing flies (Fig. 6G). RING assays showed improved climbing ability in both sexes, with males displaying a greater enhancement, as more flies reached zones 4 and 5 compared to females (Fig. 6H). Western blot analysis further confirmed that SMOX overexpression significantly reduced α-Syn protein levels (Fig. 6I). This dual outcome, in which both suppression and overexpression of SMOX attenuate α-Syn toxicity, was unexpected and suggests a more nuanced role for SMOX in regulating PA metabolism and α-Syn homeostasis. Collectively, these findings support the conclusion that overexpression of either SAT1 or SMOX mitigates α-Syn toxicity in *Drosophila* models.

## Discussion

**Figure 7:**
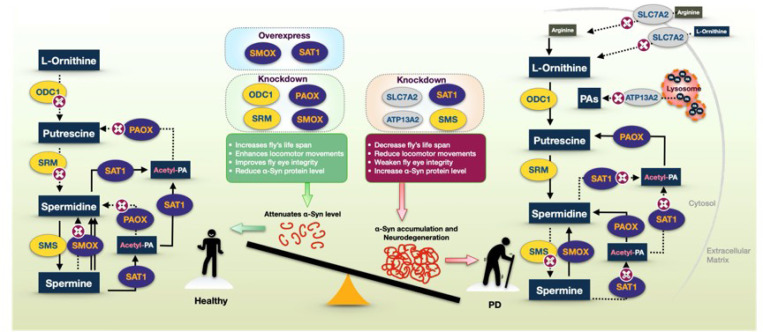
Proposed model of the polyamine pathway and its regulation of α-Synuclein levels and toxicity Illustration of the PA pathway, emphasizing how the regulation of PAIE through RNAi knockdown (indicated by 'RNAi' in rectangles or magenta X-circles in the flow diagrams) or overexpression (ovals) affects α-Syn protein levels and toxicity. The model highlights the influence of individual enzymes on polyamine metabolism, α-Syn accumulation, fly lifespan, motility, and eye integrity. The left half represents beneficial polyamine metabolism, where knockdown of ODC1, SRM, SMOX, or PAOX, or overexpression of SMOX or SAT1, reduces α-Syn levels and toxicity, promoting health and longevity. In contrast, the right half illustrates a PD-like condition, where knockdown of SMS, SAT1, ATP13A2, or SLC7A2 leads to either α-Syn accumulation or increased toxicity, ultimately resulting in neurodegeneration.

In this study, we systematically investigated the role of PA pathway enzymes in modulating α-Syn-induced toxicity using an intact organism model. Previous reports of elevated L-ORN-derived PAs in the serum of PD patients^34^ suggest a potential systemic disruption in PA metabolism. Given the tightly regulated nature of PA homeostasis^79^, we hypothesized that altered concentrations of the various PAs contribute to PD pathology, as proposed in prior studies^55,80^. To test this, we examined the functional relevance of the PA pathway in a *Drosophila* model of α-Syn toxicity, with a particular focus on PAIEs and PA transporters. Our findings demonstrate distinct phenotypic outcomes associated with specific gene knockdowns, highlighting the importance of PA metabolism in synucleinopathy and its potential as a therapeutic target (Fig. 7).

The α-Syn toxicity readouts in knockdown experiments, including longevity, motility, and GFP eye integrity assays, closely correlate with α-Syn protein levels. This highlights the functional impact of PA pathway modulation on α-Syn homeostasis and associated neurodegenerative phenotypes (Fig. 7). Among the enzymes tested, SAT1 showed the most pronounced effect. Knockdown of SAT1 exacerbated α-Syn toxicity, resulting in elevated α-Syn protein levels, increased structural degeneration in the fly eye, reduced lifespan, and impaired motility. In contrast, SMOX knockdown produced the opposite outcome, lowering α-Syn protein levels, improving eye integrity, extending lifespan, and enhancing motor performance. Notably, both SAT1 and SMOX are catabolic enzymes in the PA pathway, raising an important question: why does knockdown of each result in such divergent effects? Further investigation is needed to examine the roles of specific metabolic byproducts and the balance of individual PA species in shaping these outcomes. Suppression of ODC1 and SRM improved longevity and eye integrity, while motility was enhanced in ODC1 knockdown males and SRM knockdown flies before week 4; however, α-Syn protein levels remained unchanged. Knockdown of SMS resulted in increased α-Syn levels, yet only worsened motility and eye integrity, with no significant effect on lifespan. These findings suggest that distinct PAIEs differentially regulate α-Syn toxicity, either directly or indirectly; they highlight the complex role of PAIEs in α-Syn-associated neurodegeneration.

Our observation that both knockdown and overexpression of SMOX confer protective effects in the α-Syn *Drosophila* model was unexpected and suggests that SMOX may modulate α-Syn toxicity through multiple, potentially distinct mechanisms. SMOX catalyzes the oxidation of SPM to SPD, producing reactive oxygen species (ROS), including hydrogen peroxide (H_2_O_2_), as metabolic byproducts^61,81^’^82^, which can contribute to oxidative stress. Partial suppression of SMOX may protect neurons by limiting ROS generation, thereby reducing oxidized α-Syn-induced toxicity and genomic damage^52^. This protective effect is supported by our Western blot results showing reduced α-Syn protein accumulation, which correlates with improved cellular function as evidenced by extended lifespan, enhanced motility, and preserved neuronal integrity in the eyes. Subsequently, we observed that SMOX overexpression also produced a protective effect, which was unexpected given the benefits previously seen with SMOX knockdown. This intriguing result prompted us to test the overexpression of two copies of SMOX to determine whether a stronger effect could be achieved (data not shown). The outcome confirmed that SMOX overexpression has a protective role in our α-Syn model. Mechanistically, SMOX catalyzes the conversion of SPM to SPD, a process that may facilitate the clearance of excess SPM and restore PA balance. In addition, elevated SPD levels have been associated with various beneficial effects, including stimulation of eIF5A hypusination^83,84^, reduction of histone acetylation^85,86^, and promotion of compensatory autophagy^87–89^ and cellular repair processes^90^. These molecular changes may contribute to enhanced autophagic flux, supporting the removal of α-Syn aggregates and improving phenotypic outcomes^50^. This dual observation suggests that both reduced and elevated SMOX activity play a role in α-Syn toxicity, likely through different mechanisms.

Additionally, we found that neuronal knockdown of the PA transporters ATP13A2 and SLC7A2, as well as the catabolic enzyme PAOX, caused severe developmental abnormalities and led to early mortality in flies. These findings suggest that ATP13A2, SLC7A2, and PAOX are essential for normal developmental processes, potentially functioning both within and beyond their roles in maintaining PA homeostasis during development. Similarly, SAT1 knockdown was associated with increased α-Syn–related toxicity. As a rate-limiting enzyme in PA catabolism^62,91^, SAT1 facilitates the acetylation of SPD and SPM, allowing these PAs to be further metabolized, reintegrated into other pathways, or exported from the cell. Reduced SAT1 activity may disrupt PA flux, resulting in the accumulation of SPD and SPM, which can become cytotoxic at elevated concentrations and compromise cellular homeostasis. In addition to its enzymatic role, SAT1 also interacts with other proteins and contributes to broader cellular functions^92,93^. For example, SAT1 has been shown to bind HIF-1a and RACK1, promoting the ubiquitination and degradation of HIF-1a^94^, a transcription factor that regulates the expression of many stress- and metabolism-related genes^95,96^. Furthermore, the acetylation of PAs or other cellular targets may exert protective effects under conditions of cellular stress^59,97^. Taken together, our results suggest that SAT1 activity influences longevity, motility, and neuronal integrity in the α-Syn *Drosophila* model. The opposing outcomes observed with SAT1 knockdown versus overexpression indicate that SAT1 plays a protective role in α-Syn-induced toxicity, likely through both PA-dependent and independent mechanisms.

Overall, our study demonstrates that specific PAIEs and PA transporters significantly affect the phenotypic outcomes of α-Syn-induced toxicity in a *Drosophila* model of PD. These findings highlight the importance of PA pathway regulation in modulating α-Syn homeostasis, neuronal integrity, and disease progression (Fig. 7). Future research should focus on dissecting the underlying molecular mechanisms and evaluating whether modulation of PAIE activity can serve as a viable therapeutic strategy. Moreover, given their strong influence on disease-relevant phenotypes, PAs, PAIEs, and related transporters may also hold promise as biomarkers^98,99^ for early diagnosis and monitoring of PD^33^. Together, these insights position the PA pathway as a compelling target for both therapeutic intervention and biomarker development in synucleinopathies.

## Supplementary Material

Supplement 1

## Data Availability

Fly lines and source data are available upon request. The authors affirm that all data necessary for confirming the conclusions of the article are present within the article and figures. To request data from this study, please contact W-L. T at wtsou@wayne.edu.
