# Supplementary material for: Regulation of polyamine interconversion enzymes affects α-Synuclein levels and toxicity in a Drosophila model of Parkinson’s Disease
Source: Res Sq. 2025 May 15:rs.3.rs-6648986. Preprint. [Version 1] doi: 10.21203/rs.3.rs-6648986/v1 (PMC12136214; doi:10.21203/rs.3.rs-6648986/v1)
Supplement: Supplement 1 [file NIHPPRS6648986v1-supplement-1.pdf]

Table

Table 1 is available in the Supplementary Files section.

Supplementary Files

This is a list of supplementary files associated with this preprint. Click to download.

- Tables.docx
